# Combination therapy of simvastatin and 5, 6-dimethylxanthenone-4-acetic acid synergistically suppresses the aggressiveness of B16.F10 melanoma cells

**DOI:** 10.1371/journal.pone.0202827

**Published:** 2018-08-23

**Authors:** Valentin-Florian Rauca, Emilia Licarete, Lavinia Luput, Alina Sesarman, Laura Patras, Paul Bulzu, Elena Rakosy-Tican, Manuela Banciu

**Affiliations:** 1 Department of Molecular Biology and Biotechnology, Faculty of Biology and Geology, Babes-Bolyai University, Cluj-Napoca, Romania; 2 Molecular Biology Centre, Institute for Interdisciplinary Research in Bio-Nano-Sciences, Babes-Bolyai University, Cluj-Napoca, Romania; Rutgers University, UNITED STATES

## Abstract

The major drawback of current anti-angiogenic therapies is drug resistance, mainly caused by overexpression of the transcription factor, hypoxia-inducible factor 1α (HIF-1α) as a result of treatment-induced hypoxia, which stimulates cancer cells to develop aggressive and immunosuppressive phenotypes. Moreover, the cancer cell resistance to anti-angiogenic therapies is deeply mediated by the communication between tumor cells and tumor-associated macrophages (TAMs)—the most important microenvironmental cells for the coordination of all supportive processes in tumor development. Thus, simultaneous targeting of TAMs and cancer cells could improve the outcome of the anti-angiogenic therapies. Since our previous studies proved that simvastatin (SIM) exerts strong antiproliferative actions on B16.F10 murine melanoma cells via reduction of TAMs-mediated oxidative stress and inhibition of intratumor production of HIF-1α, we investigated whether the antitumor efficacy of the anti-angiogenic agent—5,6-dimethylxanthenone-4-acetic acid (DMXAA) could be improved by its co-administration with the lipophilic statin. Our results provide confirmatory evidence for the ability of the combined treatment to suppress the aggressive phenotype of the B16.F10 melanoma cells co-cultured with TAMs under hypoxia-mimicking conditions *in vitro*. Thus, proliferation and migration capacity of the melanoma cells were strongly decelerated after the co-administration of SIM and DMXAA. Moreover, our data suggested that the anti-oxidant action of the combined treatment, as a result of melanogenesis stimulation, might be the principal cause for the simultaneous suppression of key molecules involved in melanoma cell aggressiveness, present in melanoma cells (HIF-1α) as well as in TAMs (arginase-1). Finally, the concomitant suppression of these proteins might have contributed to a very strong inhibition of the angiogenic capacity of the cell co-culture microenvironment.

## Introduction

Melanoma cells, as well as normal melanocytes are under persistent oxidative stress due to their specialized function of melanin synthesis that generates reactive oxygen species (ROS) mainly due to the catalytic activity of tyrosinase, the rate-limiting enzyme of melanogenesis [1–3]. Moreover, in melanoma cells, melanogenesis can regulate the expression of the transcription factor, hypoxia-inducible factor 1α (HIF-1α), the key molecule for the cancer cell resistance to anti-angiogenic therapies, decreasing the effectiveness of the treatments and the survival rates during stages III and IV of melanoma [4–7]. Therefore, efforts to target melanoma angiogenesis suggested that the remaining cancer cells developed scavenger mechanisms, such as overexpression of HIF-1α, as a result of hypoxia and oxidative stress generated by these treatments. These mechanisms lead to enhanced invasive capacity of the tumor cells [8].

Moreover, the resistance of melanoma cells to anti-angiogenic therapies is also deeply mediated by the communication between tumor cells and tumor microenvironmental cells [9]. Therefore, the perspective of synergistic therapies targeting multiple microenvironment stromal cells has led to the emergence of the widely accepted “targeted microenvironment therapy” concept, rooted in the ideea that an overall “healthy” state of the microenvironment can protect against tumorigenesis and invasion [10].

Among tumor microenvironmental cell types, tumor-associated macrophages (TAMs) play a central role in supporting melanoma cell survival [11, 12], being recruited to the hypoxic tumor areas and “educated” by tumor cells [13–15] via paracrine signals (interleukin (IL)-4, IL-13, macrophage-colony stimulating factor (M-CSF), eotaxin, and transforming growth factor- β (TGF-β)) [16, 17] that convert these initial “antitumor fighters” (M1 macrophages) to “protumor slaves” TAMs (with M2 phenotypes). Nevertheless, previous studies provided evidence that macrophages infiltrated into melanoma microenvironment might present a continuum of phenotypes between M1 and M2, dependent on tumor stage [18, 19]. Thus, TAMs synthesize a wide range of protumor molecules such as pro-angiogenic and pro-inflammatory proteins (vascular endothelial growth factor (VEGF), basic fibroblast growth factor (bFGF), insulin-like growth factors (IGF), matrix metalloproteinases (MMPs), tumor necrosis factor α (TNF-α), IL-1, IL-6, IL-8, IL-10, IL-12p40, monocyte chemoattractant protein-1 (MCP-1)), as well as immunosuppressive cytokines (IL-10 and TGF-β) [20]. Moreover, TAMs-induced chronic inflammation produces reactive oxygen species (ROS) that orchestrate all processes involved in tumor progression such as angiogenesis, immunosuppression, cell proliferation, and metastasis [20].

Nevertheless, due to the advantage of high phenotypic plasticity of macrophages, TAMs can also be “re-educated” to treat cancer via therapeutic strategies that convert TAMs to antitumor macrophages [16, 17].

All these findings led to the hypothesis that targeting of TAMs might annihilate main supporting processes for melanoma development and could be a valuable strategy for improving therapeutic index of the tumor cell targeting drugs. Moreover, since previous studies described a tight connection between the antitumor efficacy of the anti-angiogenic agent—5,6-dimethylxanthenone-4-acetic acid (DMXAA) and its ability to re-activate antitumor functions of TAMs [21, 22], the present article aimed to investigate whether the antitumor efficacy of this drug could be improved by its co-administration with simvastatin (SIM)—a lipophilic statin that exerts strong antiproliferative actions on B16.F10 murine melanoma cells [12, 23, 24]. Therefore, the purpose of our study was to assess whether this combined therapy can suppress the aggressiveness of melanoma cells via targeting the communication between these tumor cells and tumor microenvironmental cells (TAMs) with the final aim to create an unfavorable milieu for the settlement of the tumor cell resistance to anti-angiogenic therapy. Thus, our recent studies proved that the antitumor activity of SIM on melanoma development was exerted via strong inhibitory effects on TAMs-mediated oxidative stress and intratumor production of HIF-1α [12, 23, 24]. Additionally, the moderate anti-angiogenic actions of SIM [12, 24, 25], proposed this statin as the drug candidate for combination therapy with DMXAA, since simultaneous targeting of complementary and redundant pathways in TAMs and cancer cells could improve the outcome of the anti-angiogenic therapies.

To assess the antitumor efficacy of the combined treatment, we used an *in vitro* model for melanoma microenvironment represented by the co-culture of bone marrow-derived macrophages (BMDMs) and B16.F10 murine melanoma cells at a cell density ratio of 4:1. This ratio provides the optimal cytokine interplay between tumor cells and macrophages, which is necessary for the approximation of murine melanoma development conditions *in vivo* [20, 26]. Furthermore, to mimic an aggressive melanoma microenvironment triggered by the “angiogenic switch”, the constitutive expression of HIF-1α in melanoma cells [24] was enhanced by the chemically induced stabilization of this transcription factor, after incubation with cobalt chloride [27, 28].

Our data suggested that the co-administration of SIM and DMXAA has the ability to suppress the aggressive phenotype of the cancer cells, as inhibitory actions on tumor cell proliferation and migration were noted. The anti-oxidant action of the combined treatment, as a result of the increase in melanin production, triggered the suppression of key molecules involved in tumor progression (HIF-1α levels in tumor cells and arginase-1 (ARG-1) levels in TAMs) and contributed to a very strong inhibitory effect on the angiogenic capacity of the cell co-culture microenvironment.

## Materials and methods

### Cell types and culture conditions

B16.F10 murine melanoma cells (ATCC, CRL-6475) were cultured in Dulbecco’s Modified Eagle’s medium (DMEM, Lonza, Basel, CH), supplemented with 10% heat-inactivated fetal bovine serum, 100 IU/ml penicillin, 100 μg/ml streptomycin and 4mM L-glutamine as monolayer at 37°C in a 5% CO_2_ humidified atmosphere.

Experiments regarding the obtaining of tumor-associated macrophages were carried out in strict accordance with the recommendations in the European (Directive 2010/63/EU) and national legislation (the Law 43/2014). The protocol was approved by the Committee on the Ethics of Animal Experiments of the Babes-Bolyai University (registration no. 31444/27.03.2017). Mice were euthanized using CO_2_ anoxia before bone collection, and all efforts were made to minimize the suffering. Thus, bone marrow cells were isolated by flushing the marrow from the femurs of 8-week-old male C57BL/6 mice (Cantacuzino Institute, Bucharest, RO) and differentiated in DMEM containing 10 ng/ml M-CSF (Cell Signaling Technology, MA, USA) [29]. These BMDMs were co-cultured with B16.F10 murine melanoma cells.

Moreover, to assess the re-education capacity of the combined treatment on TAMs, a monoculture of M2 macrophages, as predominant cell type subpopulation of TAMs [30], was used. Thus, on day 7 of *in vitro* culture, BMDMs were incubated with 20 ng/ml IL-4 (Cell Signaling Technology, MA, USA) for 24 h, which has previously been shown to promote the complete polarization of macrophages into TAMs [31, 32].

### Co-culture of B16.F10 cells with macrophages

After differentiation of bone marrow cells into BMDMs, these cells were harvested [33] and co-cultured with B16.F10 cells at a cell density ratio of 4:1 that approximates the physiological conditions of murine melanoma development *in vivo* [20, 26]. To mimic hypoxic intratumor levels of HIF-1α, cells were incubated for 24h with culture medium supplemented with 200 μM cobalt(II) chloride (CoCl_2_)–an established inducer of HIF-1α stabilization [28]. To validate the capacity of the cell co-culture model to mimic melanoma microenvironment, we compared the differences between the production of angiogenic proteins *in vitro* (protein production in the cell co-culture compared to the same protein production in B16.F10 cell monoculture) and the production of these proteins *in vivo* [11, 12] (in tumors with TAMs compared with tumors with depleted TAMs) (data not shown). There were no statistically significant differences between the overall variations of the production of pro-angiogenic proteins (*P* = 0.0729) as well as anti-angiogenic proteins (*P* = 0.2856) in the *in vitro* and *in vivo* experimental models.

### Preparation of drug solutions

The stock solution of 53 mM DMXAA (Selleckchem, Houston, TX, USA) was prepared in 100% dimethyl sulfoxide (DMSO) and kept frozen at –20°C. Working solutions of DMXAA (50, 100, 200 and 400 μM) were prepared directly into the culture medium.

SIM (Sigma-Aldrich, MO, USA) was dissolved in ethanol 70% to prepare stock solutions of 4.75 mM shortly before using. Working solutions (1.5, 3.5, 7 and 14 μM) were prepared directly into the culture media.

### Cell proliferation assay

To determine the effects of different treatments on B16.F10 murine melanoma cells proliferation, 1×10^3^ cancer cells/well were co-cultured with macrophages as shown above, in 96-well plates for 24 h. The range of concentrations for each drug was selected based on previous studies regarding their antiproliferative activity in melanoma cells [24, 34] and the effect of the drugs at each concentration was determined in triplicate. Cells incubated in medium with CoCl_2_ were used as controls. As controls for ethanol and DMSO toxicity, cells were incubated with each solvent at similar concentrations as those used for the preparation of either SIM or DMXAA working concentrations. The proliferative activity of the cancer cells after different treatments was tested using ELISA BrdU-colorimetric immunoassay (Roche Applied Science, Penzberg, DE) as previously described [24]. This method is based on the incorporation of the pyrimidine analogue—bromodeoxyuridine (BrdU)—instead of thymidine into the DNA of proliferating cells. The co-cultures of B16.F10 murine melanoma cells and macrophages were incubated with BrdU solution for 24 h and the culture medium was completely removed from each well. Then, cells were fixed and the DNA was denatured. A monoclonal antibody conjugated with peroxidase—anti-BrdU-POD—was added in each well to detect the incorporated BrdU in the newly synthesized cellular DNA. The antibody was removed after 1 h incubation, and the cells were washed three times with phosphate buffered saline. A peroxidase substrate (tetramethyl-benzidine) was added in each well, and the immune complexes were detected by measuring the absorbance of the reaction product at 450 nm with a reference wavelength of 655 nm.

### Cell apoptosis assay

To determine whether inhibition of cell proliferation is due to the induction of apoptosis in the co-culture of B16.F10 melanoma cells and macrophages under hypoxic conditions, Annexin V-fluorescein isothiocyanate (FITC) assay (Cayman Chemical, Ann Arbor, MI, USA) was used [23]. The principle of this protocol is based on the externalization of phosphatidylserine and phosphatidylethanolamine on the outer layer of the plasma membrane of apoptotic cells. The redistribution of the phospholipids is measured after high affinity binding to Annexin V conjugated with FITC. Thus, 1×10^4^ B16.F10 cells/well together with 4×10^4^ macrophages/well were seeded in 96-well black culture plate for 24 h, in the presence of 200 μM CoCl_2_. SIM was added in duplicate at concentrations ranging from 1.5 μM to 14 μM either alone or in combination with 100 μM DMXAA. After the incubation time, a double staining with Annexin V FITC and Propidium Iodide (PI) was performed at room temperature for 10 min as previously described [23]. Fluorescence emission was determined by using a fluorescence plate reader. The intensity of emission by apoptotic cells (Annexin V FITC-positive/PI-negative staining) was measured at 535 nm with an excitation wavelength at 485 nm, while for the necrotic cells (PI-positive staining) fluorescence was measured at 595 nm with an excitation wavelength at 560 nm. 5-Fluorouracil-treated cells (5 mg/ml for 24 h) were used as positive control for apoptosis. Cells cultured only in media were used as negative control.

### Cell migration assay

B16.F10 murine melanoma cells co-cultured with macrophages at a cell density ratio of 1:4 were seeded in 24-well plates. After 24 h, the confluent cell monolayers were wounded by a plastic tip (1 mm) as shown previously [35]. To remove the debris, each well was washed three times with PBS and cells were further incubated in new medium containing each treatment condition. Cells were monitored under a microscope equipped with photo camera at time 0 (time of scratching) and at 24 h after scratching. The effect of each treatment on the tumor cell migration was determined in triplicate. Images of four fields /well were recorded and analyzed by using Image J software 2016, averaging the position of the migrating cells at the wound edges [35].

### Preparation of cell lysates

The adherent co-cultured cells after different treatments were detached and lysed with lysis buffer containing 10 mM HEPES (pH 7), 200 mM NaCl, 1%Triton X, 10 mM MgCl_2_, 1 mM dithiothreitol (DTT), and protease inhibitor cocktail tablets (Complete, Roche Diagnostics GmbH, DE). The homogenates were incubated for 30 min on ice and then centrifuged for 10 min at 15 000 × *g*, at 4°C. The supernatants were collected and stored at -80°C for molecular investigations. The Bradford assay was used to determine the protein concentration of each sample (BioRad, Hercules, CA, USA) [23].

### Quantification of malondialdehyde (MDA) by HPLC analysis

MDA levels in cell lysates were determined by high-performance liquid chromatography (HPLC) as shown previously [31]. After cell lysates deproteinization, quantification of MDA was performed using HPLC column type RP18 (5 μm) (Supelco, Bellefonte, PA, USA) and a mobile phase consisting of 30 mM KH_2_PO_4_/methanol in a volume ratio of 65:35. Flow rate was set at 0.5 ml/min and MDA absorbance was measured at 254 nm. The retention time of MDA was about 5.4 min. Data were expressed as μmoles of MDA/mg of protein. Each sample was determined in duplicate.

### Determination of melanin content in B16.F10 cells co-cultured with macrophages

The melanin content of the adherent co-cultured cells after different treatments was determined according to [36, 37]. Cells were centrifuged at 400 × *g* for 5 min, dissolved in NaOH solution (1M, in 10% DMSO) and incubated at 60°C for 1 h. This step was followed by a new centrifugation at 3000 × *g* for 10 min, and melanin was detected by measuring the absorbance of the resulting supernatant at 420 nm.

### Western blot analysis of HIF- 1α levels

To determine the effect of the combined treatment based on the administration of 3.5 μM SIM and 100 μM DMXAA *versus* administration of each drug alone on the level of HIF-1α, western blot analysis was performed as described previously [23]. Thus, 15 μg of total protein were loaded per lane onto a 10% polyacrylamide gel. Electrophoresis was performed at 100 mV and then the protein fractions were electro-transferred onto a nitrocellulose membrane at 100 mV for 60 min. The membranes were blocked with 5% skimmed milk powder (BioRad, Hercules, CA, USA) in Tris-buffered saline containing 0.1% Tween-20 (TBS-T) for 2 h. Next, the membranes were incubated overnight at 4°C with rabbit monoclonal IgG against mouse HIF-1α (Abcam, Cambridge, UK), diluted 500-fold in TBS-T containing 5% skimmed milk powder. For loading control, primary rabbit polyclonal IgG against mouse β-actin (Sigma-Aldrich, MO, USA), diluted 1000-fold in TBS-T containing 5% skimmed milk powder were used. Prior to analysis, membranes were washed 4×10 min with TBS-T and incubated with goat anti-rabbit IgG HRP-conjugated (Santa Cruz Biotechnology, Dallas, TX, USA) diluted 4000-fold in TBS-T containing 5% skimmed milk powder at room temperature for 60 min. Proteins were detected using Clarity^™^ Western ECL (Bio-Rad, Hercules, CA, USA) and the membranes were exposed to an X-ray film (Kodak, Knoxville, TN, USA) for 45 s. The films were developed and analyzed using open source Image J software 2016. Transcription factor levels in each condition were compared to the same transcription factor production in control cells. The final results represented mean ± SD of two independent experiments.

### Angiogenic protein array

To assess whether the tested drugs administered alone or in combination could affect the angiogenic and inflammatory capacity of the cell co-culture, a screening for 24 proteins involved in both protumor processes was performed using the RayBio^®^ Mouse Angiogenic protein Antibody Array membranes 1.1 (RayBiotech Inc., Norcross, GA, USA) as previously described [38]. The array membranes were incubated with 200 μg of total protein in cell lysates, overnight at 4°C. A mixture of biotin-conjugated antibodies against the same angiogenic proteins as the array antibodies was added on the membranes and incubated for 2 h at room temperature, followed by incubation with HRP-conjugated streptavidin for additional 2 h. Each incubation step was followed by five washing steps. Thereafter, the membranes were incubated with a mixture of two detection buffers for 2 min, exposed to an X-ray film (Kodak, NY, USA) for 4 min and then the films were developed. The protein expression level was quantified by measuring the intensity of the color of each spot on the membranes, in comparison to the positive control spots already bound to the membranes, using TotalLab Quant Software version 12 for Windows. Each protein level from the drug treated groups was expressed as percentage of the same protein level from the untreated cells (controls). For each experimental group, the protein expression level was determined in duplicate.

### RT- qPCR quantification of TAMs markers expression

To assess whether the combined treatment based on 3.5 μM SIM and 100 μM DMXAA under hypoxia-mimicking conditions could affect the expression of two marker genes for TAMs, we performed quantitative reverse transcription PCR (RT-qPCR) for IL-10 mRNA and ARG-1 mRNA [30]. Total RNA was isolated after 24 h using an RNA kit (peqGOLD Total RNA Kit, PeqLab, Erlangen, DE). Untreated cells, incubated under hypoxia-mimicking conditions were used as controls. To avoid contamination with genomic DNA, 1 μg of total RNA was digested with 1U of RNase free DNase (Thermo Scientific, MA, USA) for 30 min at 37°C followed by addition of EDTA and incubation at 65°C for 10 min. After DNase digestion, 750 ng of total RNA was revers-transcribed into cDNA with Verso cDNA kit (ThermoScientific, MA, USA) according to manufacturer’s instructions. Identical samples from each experimental group were processed in the absence of reverse transcriptase and served as controls for genomic DNA contamination. Reverse transcription products (1μl) were added to a 25-μl reaction mix containing 1×Maxima SYBR Green qPCR Master Mix (Thermo Scientific, MA, USA) and 0.3 μM of each primer. Real-time PCR reactions were performed on a Corbett RotorGene instrument under the following cycling parameters: pre-incubation at 95°C for 10 min, cycling: 95°C for 15 s, 60°C for 30 s, and then 72°C for 30 s. To check for the primers specificity, melting curves were generated. Primers for mouse *il-10* were forward 5′- GGT TGC CAA GCC TTA TCG GA-3′ and reverse 5′-ACC TGC TCC ACT GCC TTG CT-3′. Primers for mouse *arg-1* were forward 5′- CTC CAA GCC AAA GTC CTT AGA G -3′ and reverse 5′- AGG AGC TGT CAT TAG GGA CAT C-3. Primers for the housekeeping gene mouse *β-actin* were forward 5′- TCT TTG CAG CTC CTT CGT TGC CGG TCC-3′ and reverse 5′-GTC CTT CTG ACC CAT TCC CAC CAT CAC AC-3′. Gene expression was calculated by relative quantitation using the comparative Ct method (ΔΔCt), as previously described [39]. Mouse *β-actin* mRNA was used as a reference gene expression. Gene expression was reported as fold change (2^-ΔΔCt^), relative to untreated control cells, used as calibrator.

### Determination of nitric oxide metabolites in TAMs

To investigate whether the combined treatment could affect the production of nitric oxide (NO) in IL-4-treated BMDMs, incubated under hypoxia-mimicking conditions, the levels of nitrite as the final stable product of NO metabolism were determined, as previously described [12]. Data were expressed as nmoles of nitrite/mg protein. Each sample was determined in duplicate.

### Statistical analysis

Data from different experiments were indicated as mean ± standard deviation (SD). The IC_50_ values of different treatments were calculated by using non-linear regression of sigmoidal dose response curves offered by the GraphPad Prism version 6 for Windows, GraphPad Software (San Diego, CA, USA). Two-way ANOVA with Bonferroni correction for multiple comparisons was used for the statistical analysis of the antiproliferative effects of SIM administered alone and in combination with DMXAA on B16.F10 melanoma cells as well as for the estimation of the treatments actions on angiogenic protein production. Comparisons of different treatments actions on the levels of melanin and specific markers for protumor processes were assessed by one-way ANOVA with Bonferroni correction for multiple comparisons. A value of *P*< 0.05 was considered significant.

## Results

### Synergistic action of SIM and DMXAA on murine melanoma cell proliferation

**Fig 1:**
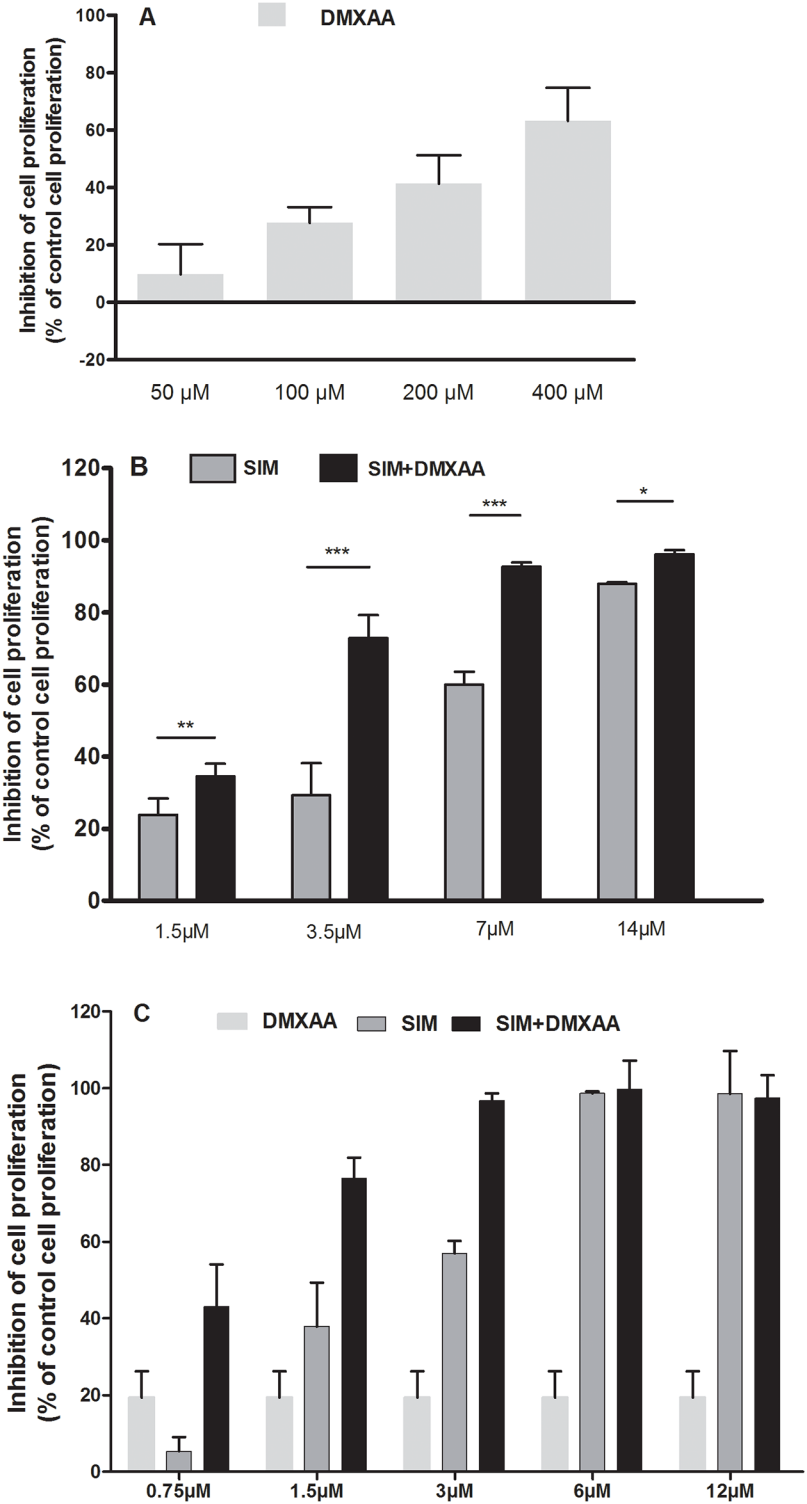
Effects of the combined administration of SIM and DMXAA on B16.F10 cell proliferation under hypoxia-mimicking conditions. (A) 24 h after incubation of co-culture of B16.F10 cells and TAMs with different concentrations of DMXAA; (B) 24 h after incubation of co-culture of B16.F10 cells and TAMs with different concentrations of SIM administered alone or in combination with 100 μM DMXAA; (C) 24 h after incubation of mono-cultured B16.F10 cells with 100 μM DMXAA administered alone and with different concentrations of SIM administered alone or in combination with 100 μM DMXAA. Data are shown as mean ± SD of triplicate measurements; DMXAA: cells incubated with different concentrations of DMXAA; SIM: cells incubated with different concentrations of SIM; SIM+ 100 μM DMXAA: cells incubated with different concentrations of SIM administered in combination with 100 μM DMXAA; The two-way ANOVA Multiple Comparison Test with Bonferroni post-tests was used to compare overall effects of different drug concentrations (*, *P*<0.05; **, *P*<0.01; ***, *P*<0.001).

The effects of different treatments on the proliferation of B16.F10 cells in monoculture and in the presence of TAMs under hypoxia-mimicking conditions were expressed as percentage of inhibition compared to the proliferation of the untreated cells (control cells) (Fig 1A–1C) and as IC_50_ values for each drug tested [40] presented in Table 1. Since the 100 μM DMXAA was the first concentration that inhibited moderately B16.F10 cell proliferation in co-culture (by 30% compared to the proliferation of control cells) (Fig 1A), this concentration was selected for combination treatments with different concentrations of SIM (Fig 1B). Notably, the association of 100 μM DMXAA with every SIM concentration tested enhanced statistically significantly the antiproliferative effects of SIM on monocultured, as well as co-cultured tumor cells (Fig 1B and 1C). Moreover, the IC_50_ of SIM decreased 1.8 times when the statin treatments were administered in combination with 100 μM DMXAA on the co-cultured cells (Table 1). To assess the nature of the interaction between SIM and DMXAA on B16.F10 cells in the presence of TAMs, the Chou-Talalay method was used to calculate the combination index (CI) [41, 42]. Thus, CI = 1 is indicative of additivity, while CI<1 or CI>1, indicates synergism or antagonism between the drugs. Our results suggested synergistic antiproliferative effects for SIM and DMXAA (CI = 0.77) (Table 1). Since 3.5 μM was the lowest concentration of SIM which administered in combination with 100 μM DMXAA exerted strong inhibitory effects (by 73% inhibition of cell proliferation compared with untreated control cells) on B16.F10 cell proliferation (Fig 1B), this combination was used throughout the experiments to further investigate the molecular mechanisms of the synergistic antitumor efficacy of both drugs on melanoma microenvironment model *in vitro*.

**Table 1:** Synergistic effect of the co-administered SIM and DMXAA on B16.F10 cells proliferation in the presence of TAMs.

Treatment	IC_50_	Confidence interval 95%	Combination index (CI)	
			CI value	Interpretation
SIM	4.825	1.232 to18.89	**-**	**-**
DMXAA	288.1	209.2 to 396.9	**-**	**-**
SIM+100 μM DMXAA	2.088	1.933 to 2.256	0.77	Synergism

**IC**_**50**_ represents the half maximal inhibitory concentration for the tested drugs and **CI** represents the “combination index”, which quantitatively depicts synergism (CI < 1), additive effect (CI = 1), and antagonism (CI > 1), according to Chou-Talalay method.

Moreover, the proliferation tests performed on the monocultured B16.F10 cells showed that SIM in combination with 100 μM DMXAA elicited strong antiproliferative effects at lower doses (1.5 μM) (Fig 1C) compared to the lowest concentration of SIM (3.5 μM) (Fig 1) which administered in combination with 100 μM DMXAA exerted strong inhibitory effects on B16.F10 cells co-cultured with macrophages. These results demonstrate once more that TAMs support B16.F10 cell proliferation/treatment resistance, therefore being a valid choice for the *in vitro* model in the present study. This finding is also in concordance with previous reports indicating that TAMs limit chemotherapy efficacy [20, 31].

### Assessment of apoptotic/necrotic effects of SIM and DMXAA on melanoma microenvironment model *in vitro*

**Fig 2:**
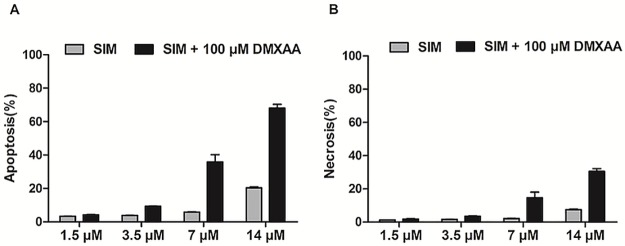
Assessment of apoptotic/necrotic effects of different concentrations of SIM administered alone or in combination with 100 μM DMXAA on the co-culture model. Double labeling for apoptosis (Annexin V-FITC) as well as for necrosis (PI) was performed. Relative fluorescence units measured were normalized for the number of cells in each condition. Data are shown as percentages of apoptosis (A) or necrosis (B) in comparison with positive controls and represented as mean ± SD of two independent measurements. SIM: cells incubated with different concentrations of SIM; SIM+100 μM DMXAA: cells incubated with different concentrations of SIM administered in combination with 100 μM DMXAA.

To investigate whether the antiproliferative action of the combined administration of SIM and DMXAA might be associated with the induction of apoptosis/necrosis in B16.F10 murine melanoma cells co-cultured with TAMs, we performed double labeling: for apoptosis with Annexin V-FITC and for necrosis with PI, as previously described [23]. The measured relative fluorescence units were normalized for the number of cells in each condition. Data were compared with positive control and shown as percentage of apoptosis and necrosis (Fig 2A and 2B). Our results indicated that only the highest concentrations of SIM (7 μM and 14 μM) administered in combination with 100 μM DMXAA exerted notable apoptotic actions on the cell co-culture (Fig 2A and 2B).

### The migration capacity of B16.F10 melanoma cells was affected by the combined treatment

**Fig 3:**
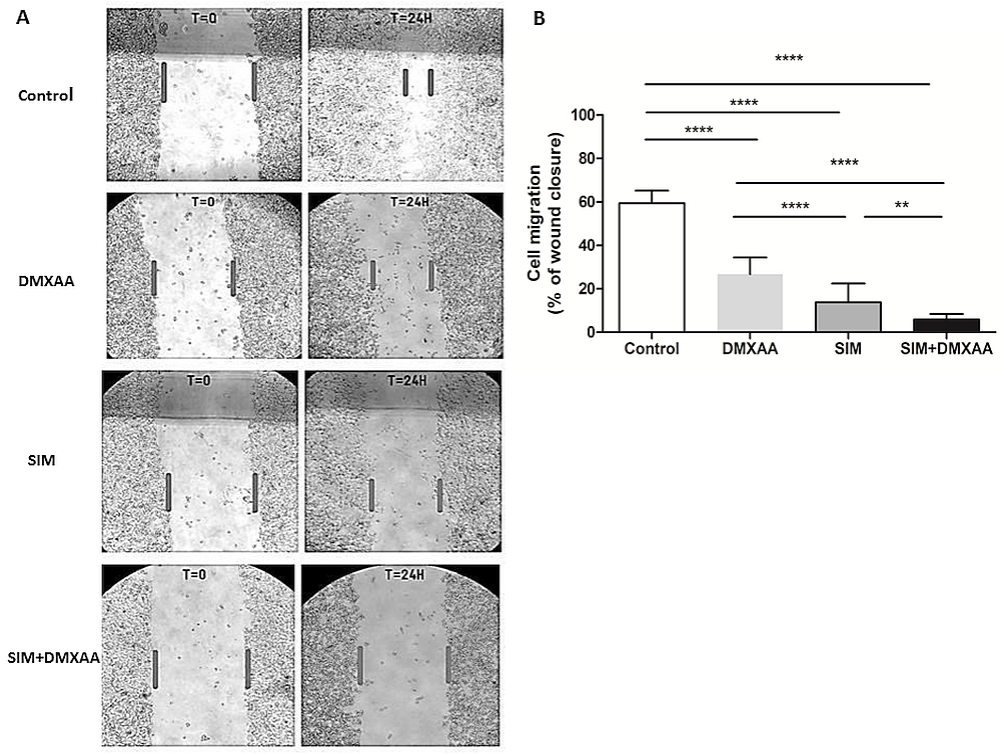
Assessment of the migration capacity of B16.F10 melanoma cells co-cultured with TAMs after different treatments. (A) B16.F10 cells co-cultured with TAMs before administration (T = 0) and 24 h after administration (T = 24H) of different treatments. Four images per well were taken for each condition (Fig 3A, magnification 100*x*). (B) The cell migration quantification. The cell migration was estimated by percentages of wound closure compared to the width of the wound at T = 0. Control = untreated cells in co-culture; DMXAA: cell co-culture incubated with 100 μM DMXAA; SIM: cell co-culture incubated with 3.5 μM SIM, SIM+DMXAA: cell co-culture incubated with 3.5 μM SIM administered in combination with 100 μM DMXAA. One-way ANOVA with Bonferroni correction for multiple comparisons was performed to determine the statistical significance (**, *P*<0.01; ****, *P*<0.0001).

To assess whether the applied treatment affected the invasive capacity of B16.F10 murine melanoma cells co-cultured with TAMs, we performed the monolayer cell migration assay (Fig 3A). Our results suggested that all treatments applied inhibited strongly tumor cell migration. Nevertheless, the suppressive effect exerted by the combined treatment was higher than those exerted by each single drug treatment (Fig 3A and 3B). Thus, the combined administration of SIM and DMXAA inhibited almost completely the migration of B16.F10 cells (more than 90% inhibition compared to the migration of untreated cells), while the administration of either DMXAA or SIM suppressed by 50–75% the invasive capacity of these cancer cells (Fig 3B). This finding suggested that the aggressive phenotype of B16.F10 cells might be affected by the tested treatments.

### Strong anti-oxidant effects of SIM and DMXAA on B16.F10 co-cultured with TAMs

**Fig 4:**
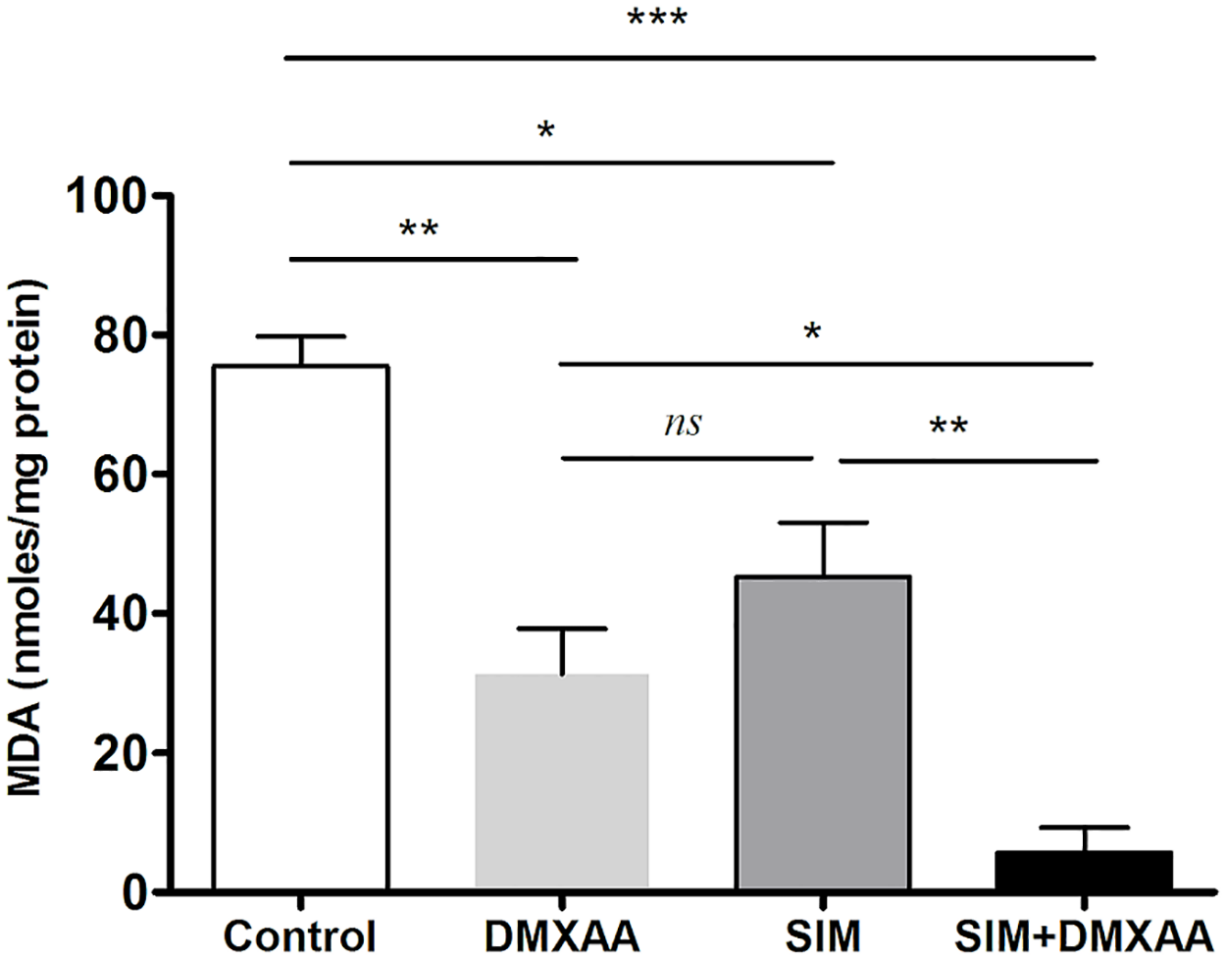
The effects of different treatments on the MDA levels from the co-culture of B16.F10 cells and TAMs, incubated under hypoxia-mimicking conditions for 24 h. The results are expressed as mean ± SD of two independent measurements. Control = untreated cells in co-culture; DMXAA: cell co-culture incubated with 100 μM DMXAA; SIM: cell co-culture incubated with 3.5 μM SIM, SIM+DMXAA: cell co-culture incubated with 3.5 μM SIM administered in combination with 100 μM DMXAA. One way ANOVA test with Bonferroni correction for multiple comparisons was performed to analyze the differences between the effects of the treatments applied on MDA levels (*ns*, *P*>0.05; *, *P*<0.05; **, *P*<0.01; ***, *P*<0.001).

To link the effects of the treatments on the cancer cell proliferation and migration to the modulation of the oxidative stress [20] in the co-culture model, we determined the levels of MDA- the lipid peroxidation product, a general marker for oxidative stress [31]. Our results showed that oxidative stress was notably suppressed by all treatments, but the strongest inhibition was caused by the combined treatment, which almost completely reduced the MDA levels in cell co-culture lysates (higher than 90% reduction compared to the production of MDA in control cells) (Fig 4). These data suggested that the very strong anti-oxidant action of the combined therapy might be responsible for the inhibition of migration capacity of these cells, but also for the suppression of B16.F10 cell proliferation presented above.

### The effects of the combined treatment on the melanin content in the cell co-culture

**Fig 5:**
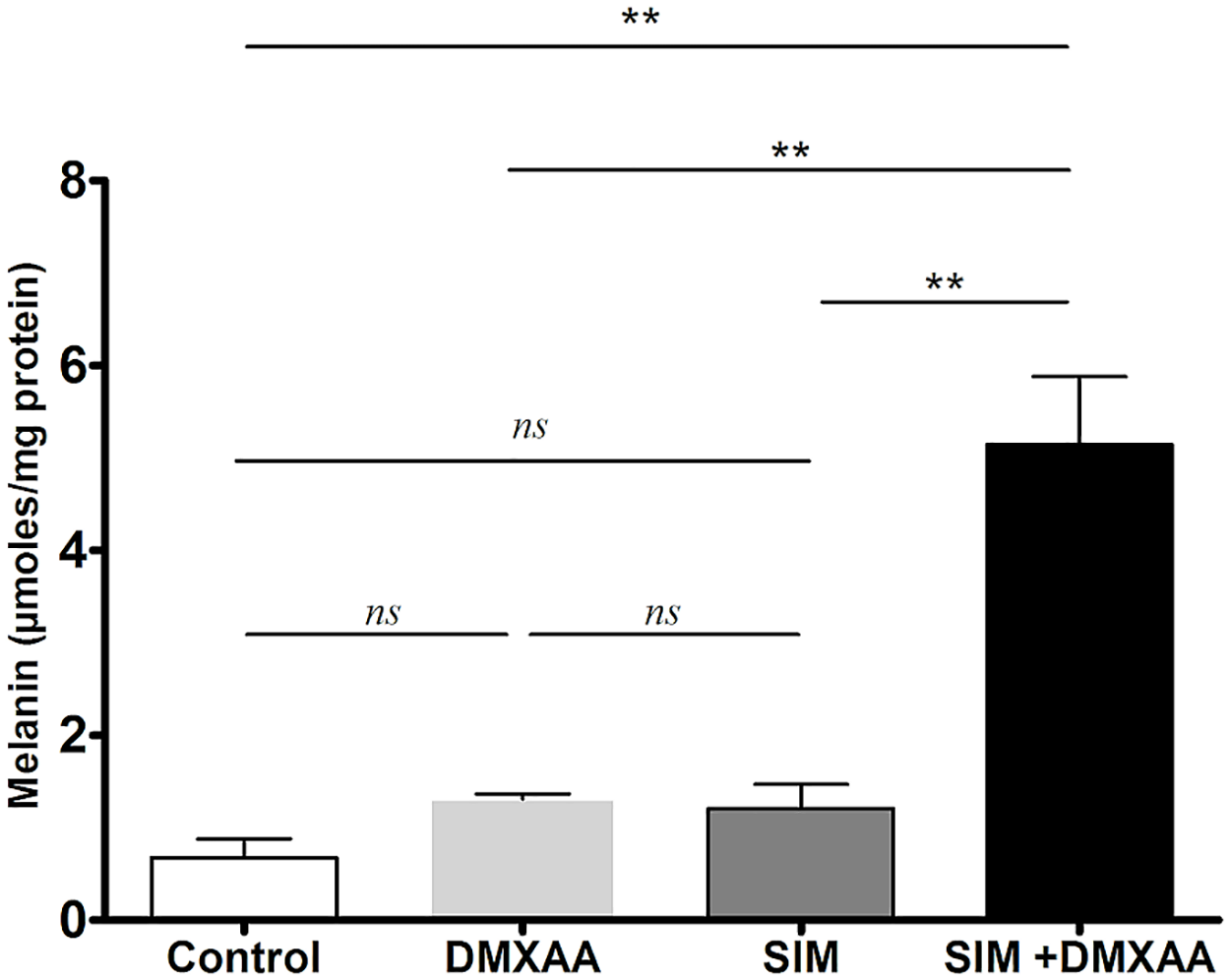
The effects of different treatments on the melanin content in the cell co-culture. The results are expressed as mean ± SD of two independent measurements. Control = untreated cells in co-culture; DMXAA: cell co-culture incubated with 100 μM DMXAA; SIM: cell co-culture incubated with 3.5 μM SIM, SIM+DMXAA: cell co-culture incubated with 3.5 μM SIM administered in combination with 100 μM DMXAA. One way ANOVA test with Bonferroni correction for multiple comparisons was performed to analyze the differences between the effects of the treatments applied on melanin content (*ns*, *P*>0.05; **, *P*<0.01).

As melanin content modulates melanoma oxidative stress [6] and the aggressive phenotype of cancer cells, the effects of the treatments on this specific pigment production in the co-culture model were assessed. Our results showed that only the combined treatment elicited the melanin production in B16.F10 melanoma cells (Fig 5). Thus, the cell co-culture incubated simultaneously with SIM and DMXAA presented a 7.5-fold higher level of melanin than in control cell co-culture (*P*<0.01).

### The combined treatment suppressed HIF-1α levels

**Fig 6:**
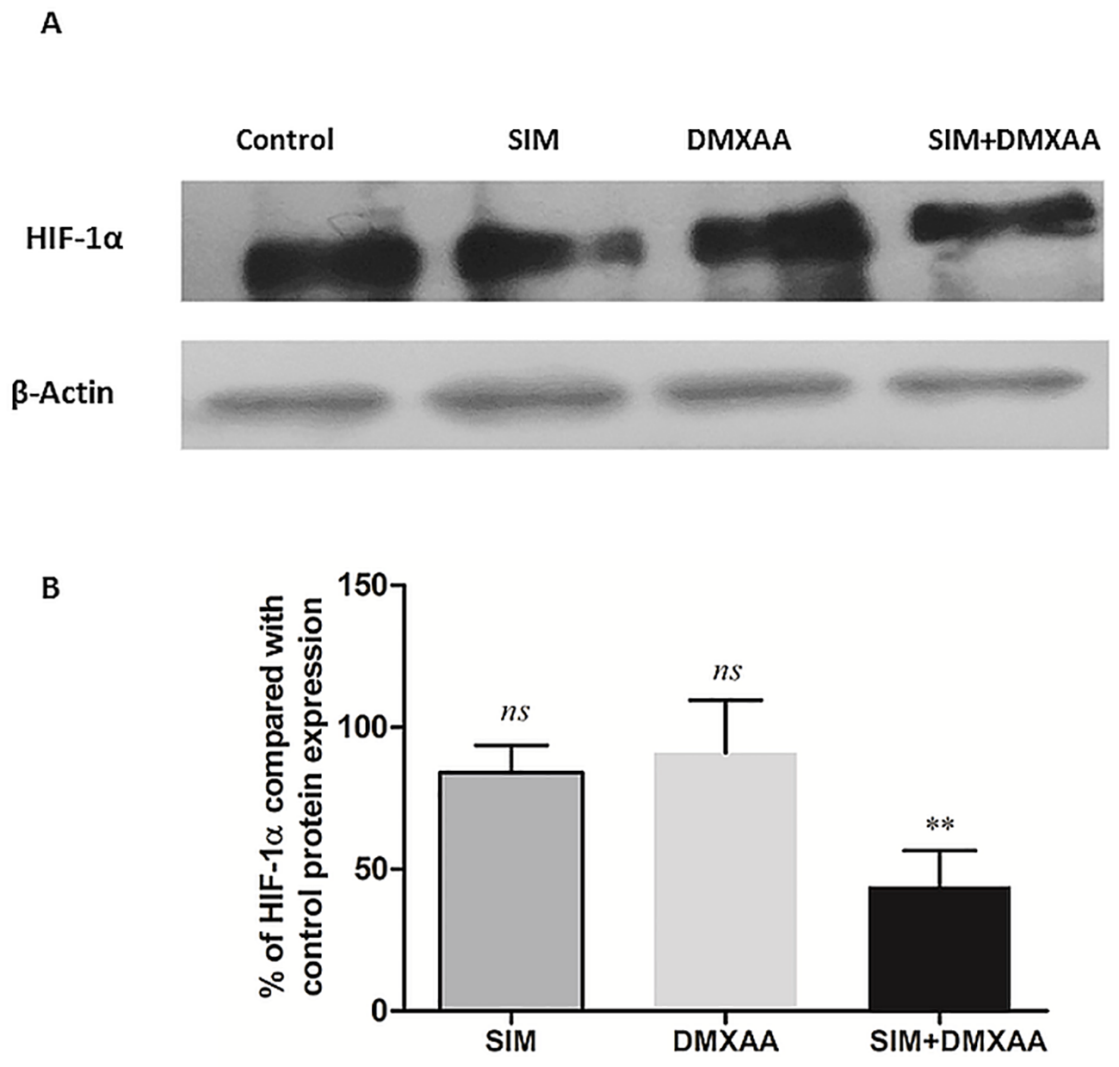
The production of HIF-1α in cell lysates after different treatments. (A) Western blot analysis of the HIF-1α production after different treatments. β-actin was used as loading control. (B) % of expression of HIF-1α in the cell co-culture after different treatments in comparison with its expression in the control (untreated cell co-culture). Control = untreated cells in co-culture; SIM: cell co-culture incubated with 3.5 μM SIM, SIM+DMXAA: cell co-culture incubated with 3.5 μM SIM administered in combination with 100 μM DMXAA. The results represent the mean ± SD of two independent measurements. One-way ANOVA test with Bonferroni correction for multiple comparisons was used to analyze the effects of different treatments on the levels of HIF-1α in comparison with transcription factor production in control (*ns*, *P*>0.05; **, *P*<0.01).

As HIF-1α overexpression is one of the main causes for the aggressiveness of cancer cells that is achieved after anti-angiogenic therapies [8], we investigated the effects of the treatments on intracellular HIF-1α production via western blot analysis. To mimic aggressive melanoma microenvironment in which the constitutive expression of HIF-1α in B16.F10 melanoma cells is enhanced by the hypoxic stabilization of this protein, the cell co-culture was incubated with CoCl_2_—a well-known inducer of this transcription factor stabilization and activation [24]. Our results showed that only the combined treatment suppressed HIF-1α expression consistently (40% reduction compared to the protein production in control cells) (*P*<0.01) (Fig 6A and 6B).

### The angiogenic capacity of cell co-culture microenvironment was strongly inhibited by the combined treatment

**Fig 7:**
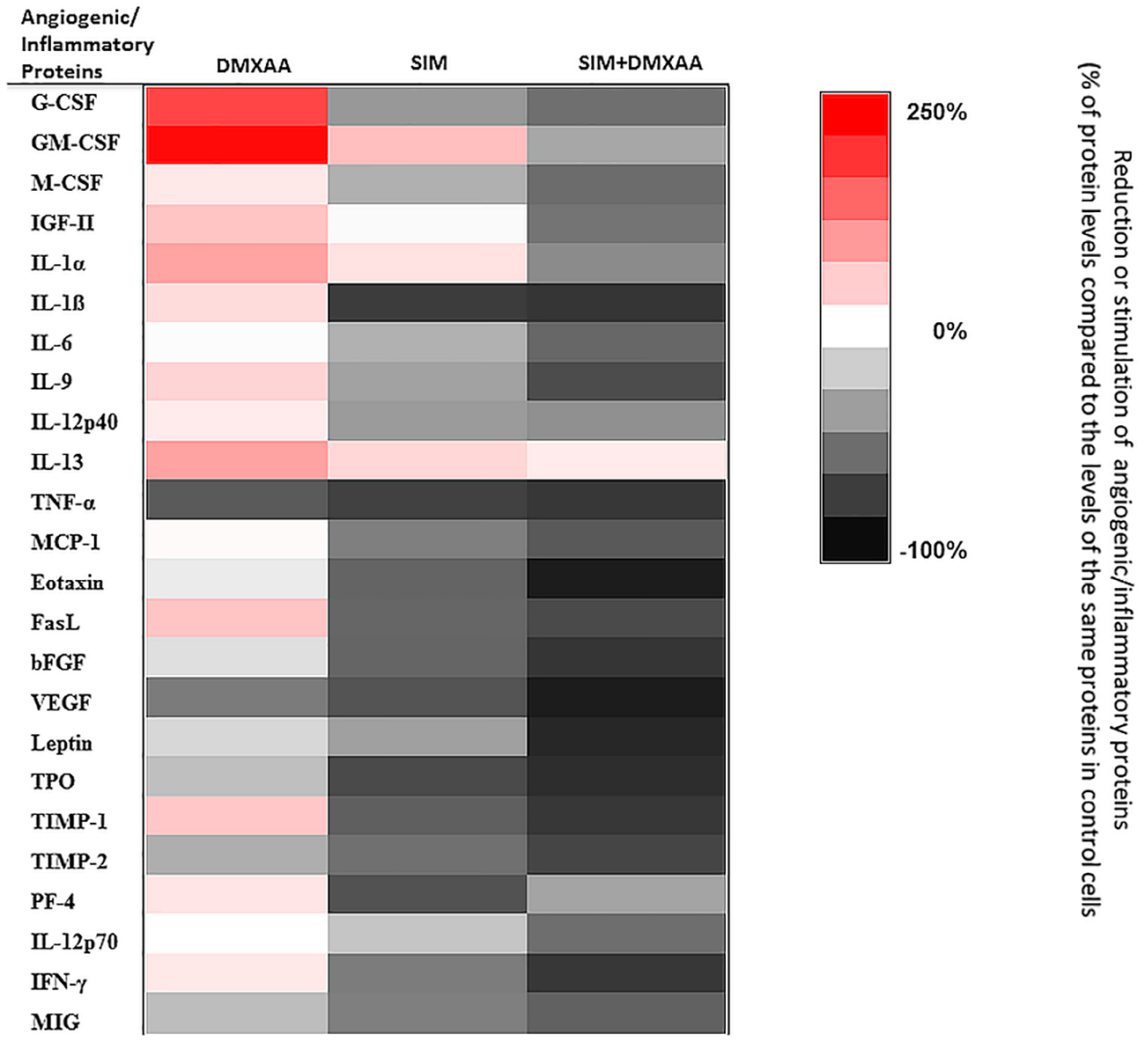
The effects of different treatments on angiogenic and inflammatory proteins production in the co-culture of B16.F10 melanoma cells and TAMs. The protein levels after different treatments are compared with the levels of the same proteins in control cells. Data are expressed as average % of reduction (-) of protein levels ranging from 0% (white) to -100% (black) or stimulation (+) of production of proteins ranging from 0% (white) to +250% (red) compared with the levels of the same proteins in control cells. DMXAA: cells incubated with 100 μM DMXAA; SIM: cells incubated with 3.5 μM SIM, SIM+DMXAA: cells incubated with 3.5 μM SIM administered in combination with 100 μM DMXAA.

To assess the effects of treatments on angiogenic capacity of the co-culture microenvironment under hypoxia-mimicking conditions, a screening for 24 angiogenic/inflammatory proteins was performed using protein array and the results are shown in Fig 7 and Tables 2 and 3. When the co-culture of B16.F10 murine melanoma cells and TAMs was incubated with 100 μM DMXAA, the average production of the inflammatory and angiogenic proteins was not significantly affected, compared to the levels in the control cells (*P* > 0.05). Only the levels of VEGF and TNF-α were significantly reduced by 55–68% (Table 2, *P*<0.05). Moreover, the production of 6 out of 24 proteins studied was stimulated moderately (50–100% enhancement of the production of insulin-like growth factor 2 (IGF-2), IL-1α, IL-13, Fas ligand (FasL), and tissue inhibitor of matrix metalloproteinases-1 (TIMP-1), compared to their control production) to strongly (150–200% stimulation of granulocyte-colony stimulating factor (G-CSF) and granulocyte-macrophage colony-stimulating factor (GM-CSF) levels compared to their control levels) (Fig 7, Tables 2 and 3). Notably, when 3.5 μM SIM was administered alone as well as in combination with 100 μM DMXAA, reduced significantly the overall intracellular production of most of the angiogenic and inflammatory proteins compared with their production in control cell co-culture. Nevertheless, the average reduction of these proteins was with 25% higher after combined treatment than after administration of SIM alone (Fig 7, Tables 2 and 3) (*P*<0.001). Thus, the levels of 14 out of 18 pro-angiogenic and pro-inflammatory proteins were drastically suppressed by 50–95% after the combined treatment. Notably, the production of all specific pro-angiogenic proteins (IGF-2, VEGF, bFGF, eotaxin, leptin, and thrombopoietin) were almost completely decelerated by 85–95% compared to their control protein production (Fig 7 and Table 2). Nevertheless, except for platelet factor 4 (PF-4), all anti-angiogenic proteins were strongly inhibited by 60–82% after cell co-culture incubation with the combined treatment (Fig 7 and Table 3).

**Table 2:** The effects of SIM and DMXAA administered as single as well as combined treatment on the pro-angiogenic protein production of the co-culture of B16.F10 cells and TAMs.

Pro-angiogenic proteins	Percentage of inhibition (-) and stimulation (+) of pro-angiogenic protein production after different treatments		
	DMXAA	SIM	DMXAA+SIM
G-CSF	+183±84 (***)	-42±4 (*ns*)	-59±8 (*)
GM-CSF	+241±126 (***)	+63± 6 (*)	-36±0.5 (*ns*)
M-CSF	+23±0.3 (*ns*)	-32±4 (*ns*)	-61±2 (*)
IGF-2	+58±2 (*)	-2±3 (*ns*)	-57±0.7 (*)
IL-1ɑ	+91±8 (***)	+29±0.9 (*ns*)	-48±6 (*ns*)
IL-1ß	-34±12 (*ns*)	-81±0.1 (***)	-83±1 (***)
IL-6	-1±42 (*ns*)	-32±8 (*ns*)	-62±5 (*)
IL-9	+43±6 (*ns*)	-38±1 (*ns*)	-74±6 (**)
IL-12p40	+20±9 (*ns*)	-41±1 (*ns*)	-46±0.5 (*ns*)
IL-13	+91±4 (***)	+40±15 (*ns*)	+20±6 (ns)
TNF-α	-68±1 (**)	-78±1 (***)	-82±1 (***)
MCP-1	+5±2 (*ns*)	-53±4 (*ns*)	-68±2 (**)
Eotaxin	-8±3 (*ns*)	-64±3 (*)	-94±2 (***)
FasL	+57±2 (*)	-63±1 (*)	-75±1 (**)
bFGF	-13±4 (*ns*)	-63±1 (*)	-84±2 (***)
VEGF	-54±0.3 (*ns*)	-71±18 (**)	-93±2 (***)
Leptin	-16±6 (*ns*)	-39±7 (*ns*)	-90±0.4 (***)
Thrombopoietin	-26±4 (*ns*)	-75±4 (**)	-87±1 (***)

The pro-angiogenic protein levels in cell lysates after different treatments are compared to control levels of the same proteins. The results are expressed as % of the average inhibition (-) or stimulation (+) ± SD of two independent measurements. The two-way ANOVA Multiple Comparison Test was used to compare overall effects of different treatments on the production of these proteins with the levels of the same proteins in controls; DMXAA: cell co-culture treated with 100 μM DMXAA; SIM: cell co-culture treated with 3.5 μM SIM; DMXAA+SIM: cell co-culture treated with 100 μM DMXAA and 3.5 μM SIM. (*ns*, *P*>0.05; *, *P*<0.05; **, *P*<0.01; ***, *P*<0.001).

**Table 3:** The effects of SIM and DMXAA administered as single as well as combined treatment on the anti-angiogenic protein production of the co-culture of B16.F10 cells and TAMs.

Anti-angiogenic proteins	Percentage of inhibition (-) and stimulation (+) of anti-angiogenic protein production after different treatments		
	DMXAA	SIM	DMXAA+SIM
TIMP-1	+56±8 (*ns*)	-66±4 (**)	-82±0.1 (***)
TIMP-2	-33±3 (*ns*)	-59±8 (*)	-77±1 (***)
PF-4	+26±21 (*ns*)	-71±6 (**)	-37±8 (*ns*)
IL-12p70	+0.2±6 (*ns*)	-24±3 (*ns*)	-60±9 (*)
IFN-γ	+23±15 (*ns*)	-54 ±4 (*ns*)	-83±1 (***)
MIG	-27±12 (*ns*)	-53±7 (*ns*)	-65±5 (**)

The anti-angiogenic protein levels in cell lysates after different treatments are compared to control levels of the same proteins. The results are expressed as % of the average inhibition (-) or stimulation (+) ± SD of two independent measurements. The two-way ANOVA Multiple Comparison Test was used to compare overall effects of different treatments on the production of these proteins with the levels of the same proteins in controls; DMXAA: cell co-culture treated with 100 μM DMXAA; SIM: cell co-culture treated with 3.5 μM SIM; DMXAA+SIM: cell co-culture treated with 100 μM DMXAA and 3.5 μM SIM. (*ns*, *P*>0.05; *, *P*<0.05; **, *P*<0.01; ***, *P*<0.001). TIMP-2—issue inhibitor of metalloproteinases 2; IFN-γ—interferon-gamma; MIG—monokine induced by interferon-gamma.

### The combined treatment partially “re-educated” TAMs

**Fig 8:**
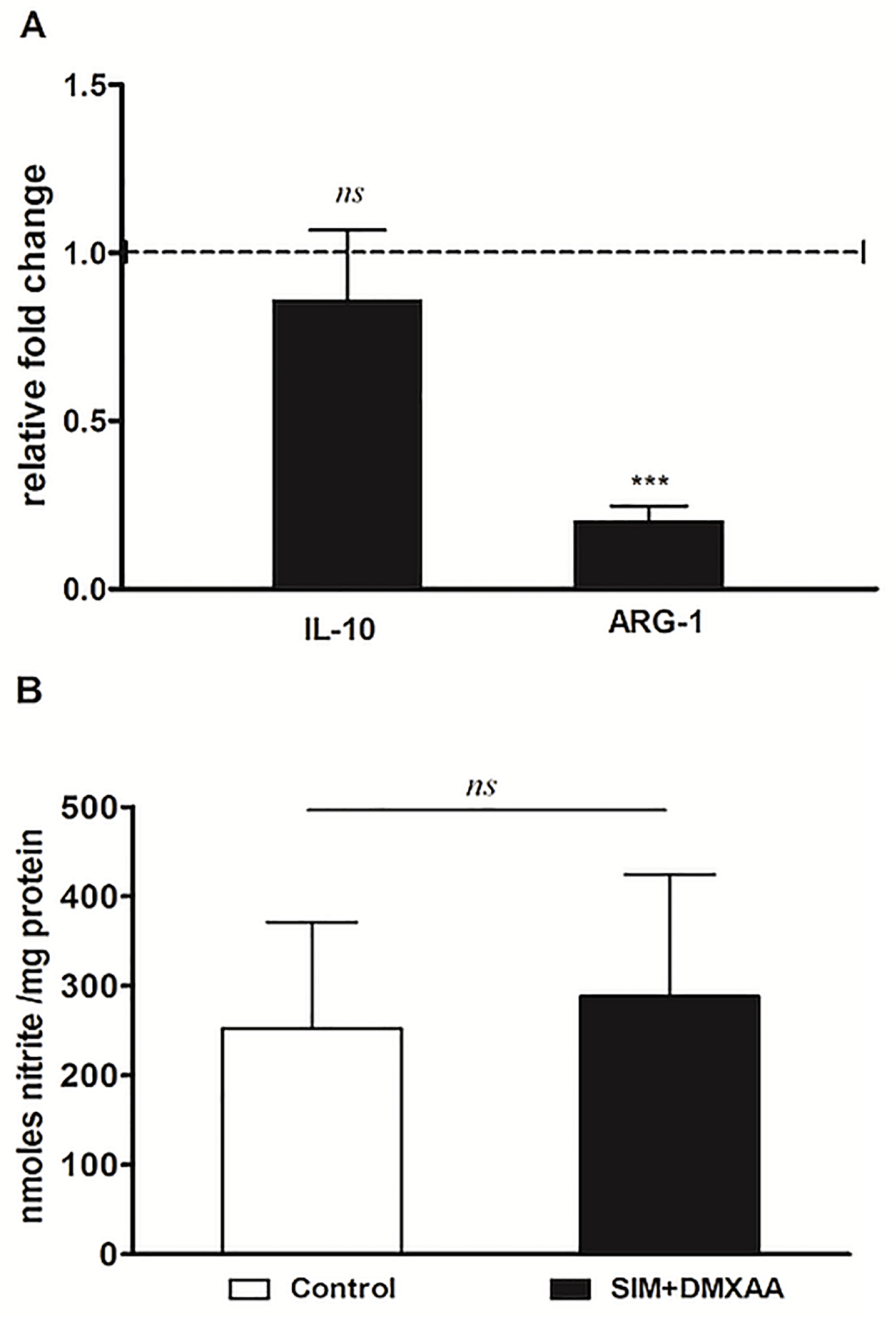
The effects of the combined treatment on macrophage polarization. (A) Effects of SIM and DMXAA co-administration on the levels of ARG-1 and IL-10 mRNA in IL-4-treated BMDMs under hypoxia-mimicking conditions. mRNA was quantified by RT-qPCR and the results are expressed as fold change based on the Ct calculations. Untreated cells were used as calibrator. The results are expressed as mean ± SD of three independent measurements (B) Nitrite levels in lysates from IL-4-treated BMDMs incubated with SIM co-administered with DMXAA under hypoxia-mimicking conditions. The results are expressed as mean ± SD of two independent measurements. Control = untreated IL-4 polarized macrophages; SIM+DMXAA = IL-4 polarized macrophages treated with 3.5 μM SIM and 100 μM DMXAA (*ns*, *P*>0.05; ***, *P*<0.001).

To investigate whether the suppressive effects on main protumor processes coordinated by TAMs could be linked to the “re-education” capacity of the combined treatment, the expression of specific markers for M2 (ARG-1 and IL-10) [30], as well as M1 phenotype (the level of nitrite–the final product of the metabolism of nitric oxide resulted from inducible nitric oxide synthase (iNOS) activity [18]) were assessed. Our data showed that only the expression level of ARG-1 was strongly reduced by 80% after TAMs incubation with the combined treatment (Fig 8A and 8B).

## Discussion

The major drawback of current anti-angiogenic therapies is the hypoxia-induced drug resistance, which stimulates cancer cells to develop aggressive and immunosuppressive phenotypes, mainly via activation of HIF-1 pathways [43, 44]. Since our previous studies reported that HIF-1α is a molecular target for SIM cytotoxicity in B16.F10 murine melanoma cells [23, 24] we investigated whether the lipophilic statin could improve the antitumor efficacy of an anti-angiogenic agent, DMXAA, when these drugs were co-administered. To our knowledge, this combined therapeutic approach has never been studied before. To mimic the aggressive melanoma microenvironment, we used the co-culture of B16.F10 murine melanoma cells and TAMs—the most powerful tumor microenvironmental cells in supporting tumor development [12]. Moreover, to enhance the constitutive expression of HIF-1α [23] in these cancer cells, the co-culture was incubated with cobalt chloride, which has the ability to stabilize HIF-1α and activate the transcription factor, HIF-1 [24, 27, 28]. Our results provided confirmatory evidence for the ability of the combined treatment to suppress the aggressive phenotype of the melanoma cells, as proliferation (Fig 1B) and migration capacity (Fig 3A and 3B) of these cells were strongly decelerated after the co-administration of SIM and DMXAA. It is noteworthy that the combination treatment was superior as antitumor efficacy to both single drug treatments tested (Figs 1A, 1B, 3A and 3B, Table 1). Nevertheless, our data suggested that the antitumor activity of the combined treatment was mainly based on cytostatic actions and not on cytotoxic effects since simultaneous administration of 3.5 μM SIM and 100 μM DMXAA in the co-culture model induced only slightly apoptosis and necrosis in these cells (Fig 2A and 2B).

To gain further evidence on the molecular mechanisms behind the antiproliferative and anti-invasive actions of the combined treatment, we assessed the level of subunit α of the transcription factor HIF-1, that is the key player for the resistance development in cancer cells to anti-angiogenic therapies [8]. Our data demonstrated that only the combined treatment suppressed significantly the production of this protein (Fig 6A and 6B). This finding might also be linked to the strong anti-oxidant activity of the combined treatment (Fig 4A) as our recent data demonstrated a tight connection between the intensity of oxidative stress and the expression and stabilization of HIF-1α in melanoma cells [12, 24]. Although previous studies have demonstrated the stimulatory role of melanin on HIF-1α expression in melanoma cells [4], it seems that enhancing effect of the combination therapy on melanin production (Fig 5) might contribute to the anti-oxidant action of SIM and DMXAA on the cell co-culture microenvironment (Fig 4) and finally, to the suppression of HIF-1α production in these tumor cells (Fig 6A and 6B). Since there are conflicting reports about the role of melanin as pro- or antioxidant, it seems that the combined treatment might favor production of eumelanin instead of pheomelanin as the last type of pigment was previously described as pro-oxidant molecule and was associated with the invasive phenotype of melanoma cells [6, 36]. Moreover, the anti-oxidant action of the combination therapy on the cell co-culture microenvironment could also be the cause of the strong reduction of ARG-1 expression in TAMs (Fig 8A), since previous studies have already proved that targeting TAMs by antioxidants reduced significantly intracellular ARG-1 activity [45]. Thus, abolishment of ARG-1 expression in TAMs, reduced the M2 response and impeded the production of polyamines, which are necessary not only for tumor cell proliferation [45, 46], but also for tumor cell invasion and metastasis [47]. This finding is also consistent with our data regarding the effects of the combined treatment on cell proliferation (Fig 1A) and migration (Fig 3A and 3B). Nevertheless, the inefficiency of SIM administered together with DMXAA on the modulation of macrophage production of NO and IL-10 (Fig 8A and 8B) might suggest the limitation of the “re-education” capacity of this treatment on TAMs. However, the lack of the M1 response with regard to iNOS activity might be beneficial for the antitumor efficacy of the combined treatment, as the increased levels of NO in macrophages would counteract its anti-oxidant action [18].

Altogether, these results demonstrated that the combined administration of SIM and DMXAA on the *in vitro* melanoma microenvironment model can target simultaneously both cell types—tumor cells and TAMs, by suppressing the expression of essential molecules (HIF-1α and ARG-1), responsible for inducing the aggressive phenotype of B16.F10 melanoma cells. Furthermore, to link inhibitory actions of the combined treatment on HIF-1α production and oxidative stress to its actions on cell co-culture potential to support vital processes for tumor development, such as angiogenesis [20, 24], we evaluated the angiogenic and inflammatory capacity of these cells after the incubation with SIM and DMXAA (Fig 7, Tables 2 and 3). Our results proved that the combined treatment exerted very strong inhibitory actions on the production of most of the pro-angiogenic proteins in the co-culture of B16.F10 murine melanoma cells and TAMs (Fig 7 and Table 2). It is noteworthy that VEGF, as primary effector molecule of HIF-1 activation of angiogenesis-related genes [48], as well as eotaxin as pro-invasive protein [49], were almost totally decelerated after the administration of the combination between SIM and DMXAA on the cell co-culture. Nevertheless, almost all anti-angiogenic proteins production was strongly inhibited after this treatment (Fig 7 and Table 3). This limitation of the combined treatment effect on cell co-culture angiogenic capacity might be linked to its inefficiency to suppress IL-10 expression in TAMs (Fig 8A). The continuous presence of immunosuppressive IL-10 in the co-culture microenvironment mediates the inhibition of one of the most important anti-angiogenic protein, IL-12, an inducer of IFN-γ production [50–52]. Therefore, a future functional association of this therapy with IL-12 could overcome the current limitations of the drug combination. However, strong suppression of IFN-γ production, together with the inhibition of pro-inflammatory cytokine expression (IL-1ß, IL-6, IFN-γ and TNF-α) (Tables 2 and 3), might also have contributed to the increase in melanin production in melanoma cells (Fig 5) [5, 53] and finally, to the enhancement of the anti-oxidant actions of the combined treatment.

In conclusion, our data demonstrated that the combined administration of SIM and DMXAA on the co-culture of B16.F10 murine melanoma cells and TAMs under hypoxia-mimicking conditions has the potential to become a successful targeted microenvironment therapy, based on the suppression of cancer cell aggressive phenotype. The molecular mechanisms behind the antitumor activity of the combined treatment involved the creation of an unfavorable microenvironment for melanoma cell proliferation and migration. Thus, the anti-oxidant action on the co-culture milieu was the principal cause for simultaneous suppression of key molecules involved in tumor progression in both cell types tested (reduction of HIF-1α levels in melanoma cells and ARG-1 levels in TAMs). As a consequence of concomitant suppression of these proteins, the angiogenic capacity of the cell co-culture microenvironment was strongly decelerated.

## Abbreviations

ANOVA: Analysis of variance
ARG-1: arginase-1
bFGF: Basic fibroblast growth factor
BMDMs: bone marrow derived macrophages
CoCl_2_: Cobalt(II) chloride
DMEM: Dulbecco’s Modified Eagle’s Medium
DMSO: Dimethyl sulfoxide
DMXAA: 5,6-dimethylxanthenone-4-acetic acid
FasL: Fas ligand
FITC: Annexin V-fluorescein isothiocyanate
G-CSF: Granulocyte-colony stimulating factor
GM-CSF: Granulocyte-macrophage-colony stimulating factor
HEPES: 4-(2-hydroxyethyl)-1-piperazineethanesulfonic acid
HIF-1α: hypoxia-inducible factor 1α
HPLC: High-Performance Liquid Chromatography
HRP: horseradish peroxidase
IFN-γ: Interferon γ
IGF-2: Insulin-like growth factor 2
IL-12 p40: Interleukin 12 p40
IL-12 p70: Interleukin 12 p70
IL-13: Interleukin 13
IL-1α: Interleukin 1α
IL-1β: Interleukin 1β
IL-6: Interleukin 6
IL-9: Interleukin 9
MCP-1: Monocyte chemoattractant protein-1
M-CSF: Monocyte-colony stimulating factor
MDA: Malondialdehyde
MIG: Monokine induced by IFN-γ
NF-κB: Nuclear factor κB
PBS: Phosphate buffered saline
PF-4: Platelet factor 4
PI: Propidium Iodide
ROS: reactive oxygen species
SD: Standard Deviation
SIM: Simvastatin
TAMs: Tumor-associated macrophages
TBS-T: Tris-buffered saline containing 0.1% Tween-20
TGF-β: transforming growth factor-β
TIMP-1: Tissue inhibitor of metalloproteinase 1
TIMP-2: Tissue inhibitor of metalloproteinase 2
TNF-α: Tumor necrosis factor α
TPO: Thrombopoietin
VEGF: Vascular endothelial growth factor
